# Molecular mechanism of internode elongation in rice

**DOI:** 10.1270/jsbbs.22086

**Published:** 2023-04-27

**Authors:** Keisuke Nagai, Motoyuki Ashikari

**Affiliations:** 1 Bioscience and Biotechnology Center, Nagoya University, Furo, Chikusa, Nagoya, Aichi 464-8601, Japan

**Keywords:** rice, internode elongation, flooding, ethylene, gibberellin

## Abstract

Rice plants that form ventilated tissues, such as aerenchyma in the leaves, stems, and roots, allow for growth in waterlogged conditions (paddy fields), but they cannot breathe and drown in flooded environments where the whole plant body is submerged. However, deepwater rice plants grown in flood-prone areas of Southeast Asia survive in prolonged flooded environments by taking in air through an elongated stem (internode) and leaves that emerge above the water surface, even if the water level is several meters high and flooding continues for several months. Although it has been known that plant hormones, such as ethylene and gibberellins, promote internode elongation in deepwater rice plants, the genes that control rapid internode elongation during submergence have not been identified. We recently identified several genes responsible for the quantitative trait loci involved in internode elongation in deepwater rice. Identification of the the genes revealed a molecular gene network from ethylene to gibberellins in which internode elongation is promoted by novel ethylene-responsive factors and enhances gibberellin responsiveness at the internode. In addition, elucidation of the molecular mechanism of internode elongation in deepwater rice will help our understanding of the internode elongation mechanism in normal paddy rice and contribute to improving crops through the regulation of internode elongation.

## Introduction

**Fig. 1.**
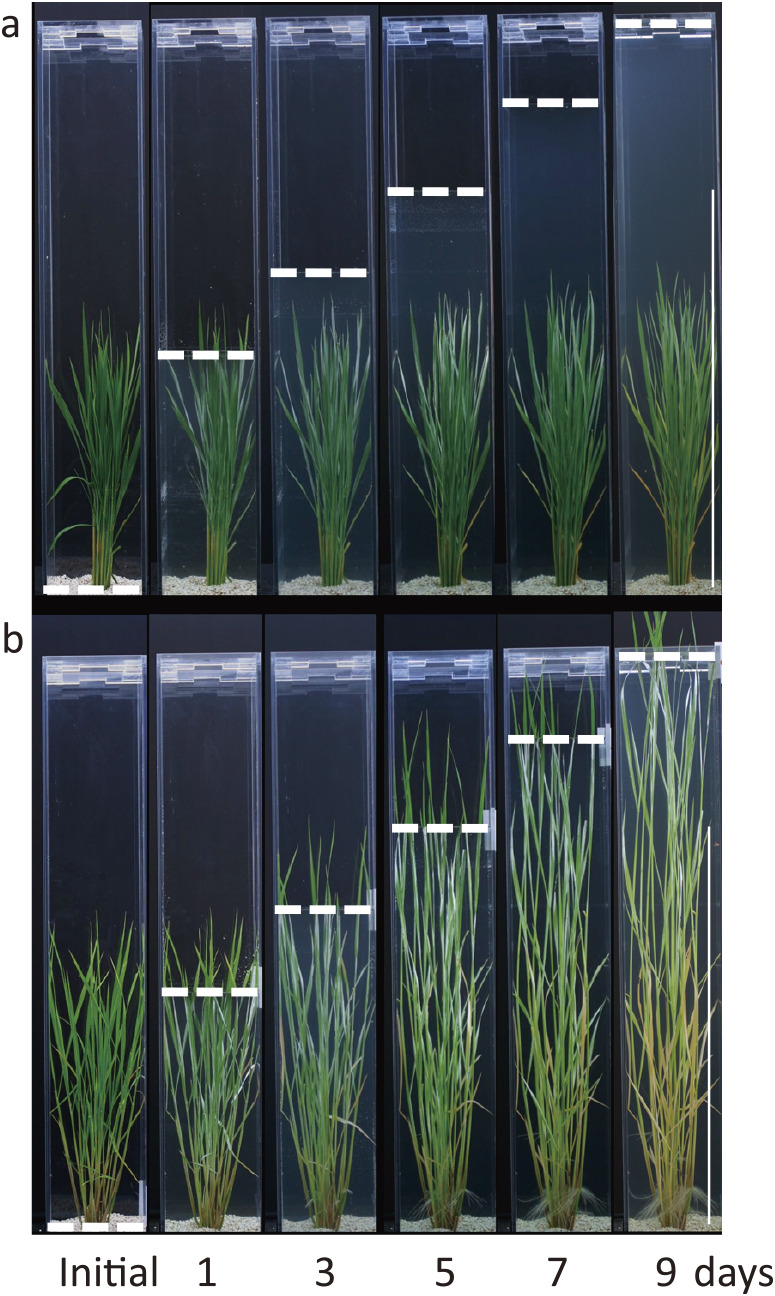
Morphological changes of rice under deep-water condition. a. Normal paddy rice (Taichung 65). b. Deepwater rice (C9285). Deepwater treatment was started at a depth of 60 cm and increased by 10 cm each day. Dashed lines and white bars represent water surface and 1m, respectively.

**Fig. 2.**
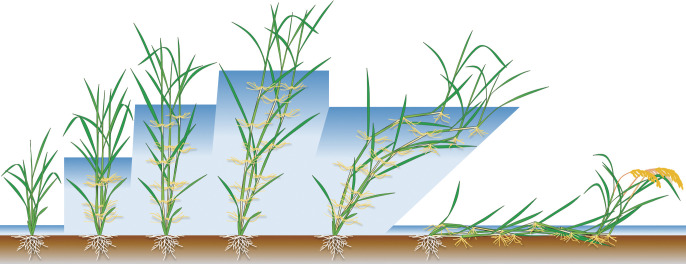
Growth of deepwater rice. The figure represents the progression of time from left to right.

Wheat, maize, and rice are the three most important cereal crops worldwide; together they provide more than 40% of the calories consumed by humans (FAO 2018). Of these, rice is the only crop that can grow in a waterlogged environment using a unique mechanism of water-tolerance. Rice leaves are water repellent (superhydrophobicity) due to wax accumulation on the leaf surface (Kurokawa *et al.* 2018, Raskin and Kende 1983). These plants supply oxygen efficiently through the leaves to the roots by developing aerenchyma aeration tissue and a radial oxygen loss barrier that prevents the leaking of oxygen in the roots (Yamauchi and Nakazono 2022). Rice can grow in a shallow water paddy field using water-tolerant mechanisms, but it cannot survive submergence. There are rice varieties that can survive in deepwater conditions, however. Deepwater rice, which is cultivated mainly in the monsoon regions of Southeast Asia, has developed a unique ability to survive in flooded environments, such as the ability to maintain respiration and photosynthesis by elongating the stem (internode) in response to rising water levels and maintaining its leaves above the water surface during flooding (Figs. 1, 2). Adventitious roots from nodes in the water are thought to facilitate the uptake of nutrients. As the flood subsides, the plant falls down, but the top of the shoot continues to face upward due to kneeing (negative gravitropism) at the upper nodes. Subsequently, when the water level decreases further, some of the adventitious roots grow into the soil and kneeing keeps the panicle above water even during the ripening stage (Fig. 2) (Catling 1992). Elucidation of the mechanism of internode elongation in deepwater rice and its application to rice breeding is a strategy to counteract crop damage caused by floods, which are expected to become more frequent due to climate change. We previously identified genes encoding ethylene-responsive transcription factors [*SNORKEL1* (*SK1*) and *SNORKEL2* (*SK2*)] and a gibberellin (GA) biosynthesis enzyme (*GA20OX2*) that promote internode elongation in deepwater rice plants. We also recently identified two genes, *ACCELERATOR OF INTERNODE ELONGATION 1* (*ACE1*) and *DECELERATOR OF INTERNODE ELONGATION 1* (*DEC1*), which regulate GA responsiveness in deepwater rice plants. *ACE1* and *DEC1* are involved in initiating internode elongation in response to GAs. In this review, we introduce the mechanism of internode elongation and the genes responsible for it in rice.

## Physiological and genetic analyses of deepwater rice internode elongation

**Fig. 3.**
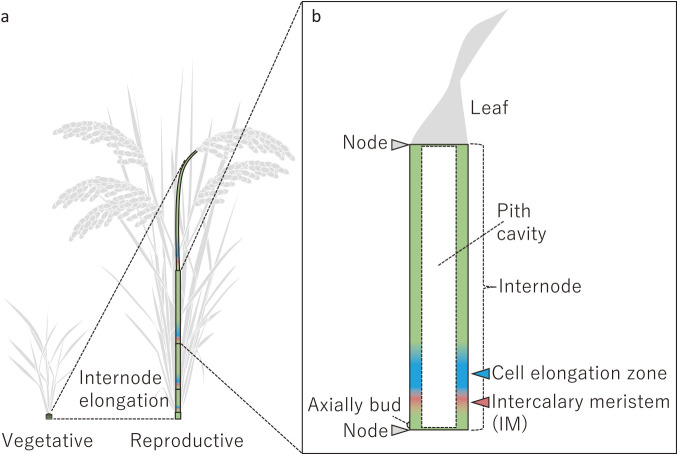
Internode elongation of rice. a. Initiation of internode elongation after phase transition. b. Phytomer of rice. The internodes elongate by cell division in intercalary meristem and subsequent cell elongation in cell elongation zone.

**Fig. 4.**
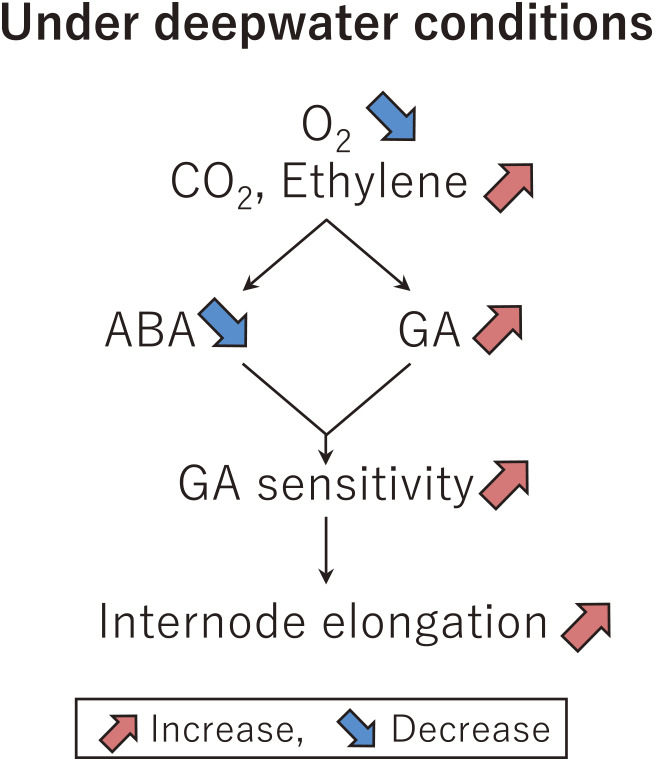
Physiological schemes in internode elongation in deepwater rice plants.

Rice consists of a series of vertically stacked component units called phytomers, which are composed of a single leaf, nodes, an internode, and an axillary bud. Rice plants do not generally elongate at the internode during vegetative growth as the internodes are packed at the base of the shoot (Fig. 3a). However, during the transition from vegetative to reproductive growth, internode elongation starts due to vertical (upward direction) cell division at the intercalary meristem (IM), which is on the basal part of the young developing internode. Subsequently, cell elongation occurs in the cell elongation zone of these cells (Fig. 3b). Activation of the IM leading to internode elongation occurs only in sequentially developing internodes after flowering. The internode elongation allows pushing the panicle formed at the stem apex to be exposed to the air for fertilization. In contrast to such a normal paddy rice, deepwater rice has acquired the ability to elongate the internode early during vegetative growth, without the phase transition from vegetative to reproductive growth. This elongation is significantly enhanced in deepwater environments, such as in floods. In addition, in normal paddy rice and deepwater rice, a pith cavity (hollow structure) forms in the center of the rice internode due to cell death. Therefore, deepwater rice plants survive in flooded environments because their leaves are exposed above the water surface through elongation of the internode to maintain respiration and photosynthesis, and the pith cavity in the elongated internode acts like a snorkel to supply oxygen to tissues below the water surface (Kende *et al.* 1998). A study of internode elongation in deepwater rice is a good model for understanding internode elongation in rice. Many studies have been performed on the physiology of deepwater rice by Dr. Kende and his colleagues at Michigan State University. The level of oxygen decreases rapidly in deepwater rice plants under flooded conditions, while the concentration of carbon dioxide increases. In addition, rice plants accumulate ethylene, a gaseous phytohormone with low diffusion in water. The accumulation of ethylene decreases the synthesis of abscisic acid (ABA), a hormone that negatively regulates internode elongation (Hoffmann-Benning and Kende 1992, Kende *et al.* 1998) (Fig. 4). In contrast, ethylene increases the synthesis and responsiveness of GA, which functions antagonistically with ABA (Hoffmann-Benning and Kende 1992) (Fig. 4). These responses are thought to promote internode elongation in deepwater rice plants. Similar phenomena were observed in our follow-up examinations (Hattori *et al.* 2009, Nagai *et al.* 2020). We have observed that ethylene or GA treatment of deepwater rice plants promotes internode elongation. Interestingly, no internode elongation was induced in normal paddy rice, even though ethylene accumulated and ABA decreased under flooded conditions. These results suggest that genetic differences in internode elongation are present beyond common ethylene accumulation and a decrease in ABA between normal paddy rice and deepwater rice.

**Fig. 5.**
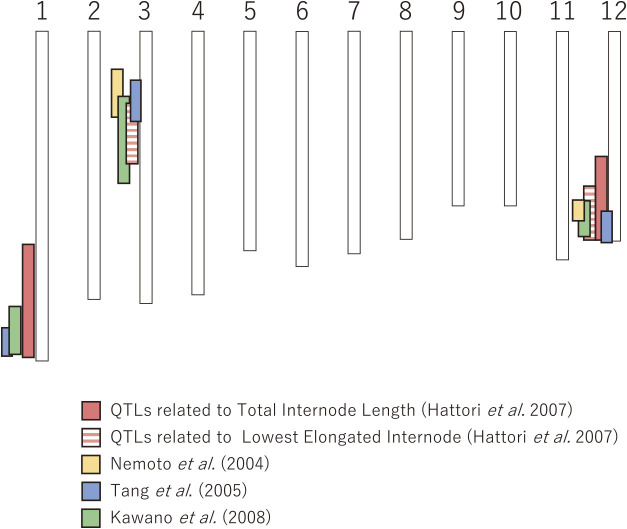
QTLs for internode elongation in deepwater rice under submerged conditions. QTLs for internode elongation were detected on chromosomes 1, 3, and 12 by four research groups. The positions of the QTLs on the chromosomes are shown in the figure with the genetic distance replaced by the physical distance.

To identify the genes regulating internode elongation in deepwater rice, we performed a quantitative trait loci (QTL) analysis for internode elongation under submerged conditions using F_2_ population of a deepwater rice line, C9285 (Dowai38/9) from Bangladesh and normal paddy rice cultivar, ‘Taichung 65 (T65)’ from Taiwan (Hattori *et al.* 2007). The results showed that *qTIL1* and *qTIL12*, which control total internode length (TIL), were detected on chromosomes 1 and 12, respectively (Fig. 5). We also detected *qLEI3* and *qLEI12*, which are regulators of the lowest elongated internode (LEI), a parameter of early internode elongation, on chromosomes 3 and 12, respectively (Fig. 5). The finding that *qTIL12* and *qLEI12* were detected on the terminal region of chromosome 12 suggested the possibility that causative genes of these QTLs might be identical, or if not, they are closely located to each other (see details below). Dr. Nemoto’s group at the University of Tokyo conducted a QTL analysis for internode elongation using the F_2_ populations of the deepwater rice varieties Habiganji Aman VII and Goai (both from Bangladesh) and normal paddy rice Patnai23 (Nemoto *et al.* 2004, Tang *et al.* 2005), and Dr. Yoshimura’s group at Kyushu University also conducted a QTL analysis for internode elongation using F_2_ populations of the deepwater rice varieties Bhadua (from Bangladesh) and T65 (Kawano *et al.* 2008) (Fig. 5). Interestingly, among the four independent QTL analyses using different rice varieties and their progenies, QTLs for internode elongation were commonly detected on chromosomes 1, 3, and 12, suggesting that these QTLs play a pivotal role in internode elongation in deepwater rice.

## Identification and functional analysis of the genes responsible for QTLs

We first performed positional cloning of *qTIL12*, which has the greatest effect on internode elongation, using cross progeny of deepwater rice variety C9285 and normal paddy rice T65, and identified two genes of the ERF family, *SK1* and *SK2*, which contain an AP2/ERF domain (Hattori *et al.* 2009). The expression of *SK1* and *SK2* was significantly induced by deepwater or ethylene treatment, and overexpression of these genes promoted internode elongation in normal paddy rice, suggesting that *SK1* and *SK2* regulate internode elongation in response to ethylene. The transcription factor ETHYLENE INSENSITIVE 3 (EIN3), which plays a central role in ethylene signaling in *Arabidopsis*, is recognized by the F-box proteins EBF1/EBF2 and subsequently degraded by the proteasome pathway in the absence of ethylene (Guo and Ecker 2003). However, EIN3 protein stabilizes due to degradation of *EBF1*/*EBF2* mRNAs by an 5ʹ→3ʹ exoribonuclease EIN5 in the presence of ethylene, resulting in an ethylene response through the upregulated expression of downstream genes (Olmedo *et al.* 2006, Van de Poel and Chang 2015). We verified whether *Oryza sativa* EIN3-LIKE 1 (OsEIL1), the rice ortholog of EIN3, binds to the promoter sequences of *SK1* and *SK2* using gel shift assays and showed that OsEIL1 bound to the respective promoters. Next, we compared genome sequences of *SK1* and *SK2* regions of deepwater and normal paddy rice. As the results, it was clarified that C9285 and another deepwater rice variety, Bhadua, retained *SK1* and *SK2*, whereas the normal paddy rice T65 and Nipponbare lacked a genomic region of about 45 kb, including *SK1* and *SK2* (Hattori *et al.* 2009, Nagai *et al.* 2022). On the other hand, the sequence very similar to the *SK* gene existed near the region where the *SK* genes were absent in normal paddy rice, but this similar sequence did not exist in the deepwater rice varieties C9285 and Bhadua (Nagai *et al.* 2022). Therefore, we named this gene *SNORKEL-LIKE 1* (*SKL1*) and examined its expression. Unlike *SK1* and *SK2*, *SKL1* expression was not increased by deepwater treatment. To investigate the function of *SKL1*, we generated *SKL1*-overexpressing plants and observed their phenotype. *SKL1* overexpressors promoted internode elongation as those of *SK1* and *SK2* in the T65 background. These results suggested that the loss of internode elongation in normal paddy rice plants was caused by a loss of the mechanism for upregulating *SKL1* in deepwater environments, in addition to a loss of the *SK* genes. The sequences of the *SK* genes were also examined in wild rice. W0120 (*Oryza* *rufipogon*), which undergoes internode elongation in deepwater environments, retained *SK1* and *SK2*, whereas W0106, the different accession of *O. rufipogon* that does not exhibit internode elongation in deepwater environments, was deficient in *SK2* due to insertion of a transposon (Hattori *et al.* 2009, Nagai *et al.* 2022). These results suggest that ethylene accumulates in the body of deepwater rice plants in a flooded environment and that this accumulation promotes internode elongation through *SK* genes whose expression is upregulated in deepwater rice plants. In contrast, although ethylene also accumulates in normal paddy rice as in deepwater rice, it does not promote internode elongation because of the absence of *SK* genes and defective expression of *SKL1*. It was recently reported that in *Arabidopsis* ERF11 regulates internode elongation by activating GA synthesis and suppressing the DELLA protein, which is a suppressor of GA signaling (Zhou *et al.* 2016). As SKs and SKL1 belong to the AP2/ERF family like ERF11, these proteins may also regulate internode elongation via GA biosynthesis or through an interaction with DELLA proteins.

We also identified *GA20OXIDASE 2* (*GA20OX2*), which encodes a GA biosynthesis enzyme, as the *qTIL1* causative gene (Kuroha *et al.* 2018). At least four *GA20OX* genes are present in the rice genome, and the proteins encoded by these genes catalyze two parallel pathways in the GA biosynthesis in the following: GA_53_ to GA_20_ and GA_12_ to GA_9_ (Yamaguchi 2008). GA_20_ and GA_9_ are converted by GIBBERELLIN 3 OXIDASE (GA3OX) to the active GA species GA_1_ and GA_4_, respectively (Yamaguchi 2008). A comparison of the amino acid sequences of *GA20OX2* from the deepwater rice variety C9285 and the normal paddy rice variety T65 showed two amino acid differences. T65 had E (glutamic acid) and Q (glutamine) at the 100th and 240th positions (EQ type), whereas C9285 deepwater rice had G (glycine) and R (arginine) (GR type) at the same positions in GA20OX2. A comparison of the enzymatic activities of the two types of proteins in GA synthesis revealed that the GR type *GA20OX2* of deepwater rice was higher than the EQ type *GA20OX2* of normal paddy rice in both catalyzing from GA_53_ to GA_20_ and from GA_12_ to GA_9_, suggesting that deepwater rice plants produce higher amounts of active GAs. As a result, deepwater rice accumulated greater amounts of GA_1_ and GA_4_ than normal paddy rice. Furthermore, the expression levels of *GA20OX2* in deepwater rice and normal paddy rice were analyzed under air and deepwater conditions. Both types of genes were slightly expressed under the air condition, but GR-type *GA20OX2* was rapidly and highly induced under the deepwater condition. In contrast, gene expression level of EQ-type *GA20OX2* did not change under the deepwater condition. As deepwater rice is known to elongate internodes even in response to ethylene treatment, we examined *GA20OX2* gene expression during ethylene treatment. The expression level of GR-type *GA20OX2* increased in deepwater rice in response to ethylene treatment, but no ethylene-induced increase in EQ-type *GA20OX2* gene expression was observed in normal paddy rice. This result suggests that signal transduction from ethylene to GA occurs in deepwater rice. Therefore, we performed a promoter analysis of the *GA20OX2* gene using OsEIL1, a key transcription factor in ethylene signaling, to determine whether OsEIL1 induces gene expression by binding to the *GA20OX2* promoter. *In vitro* analyses showed that OsEIL1 bound to the *GA20OX2* promoter in both C9285 and T65. This observation suggests the possibility that additional unknown factor to OsEIL1 is involved to upregulate deepwater rice-specific *GA20OX2* expression *in vivo*. Although further analysis is required to elucidate the detailed regulatory mechanism of *GA20OX2* expression in deepwater rice, these results suggest the existence of a molecular signal relay between ethylene and GA, such as the transfer of ethylene signaling via OsEIL1 to GA biosynthesis in deepwater rice.

Here, we outline the concept of LEI before discussing the identification of *qLEI3* and *qLEI12*. Because internode length is one of the most important agronomic traits in rice, research on internode length and strength has been conducted extensively since the 1960s. Dr. Suetsugu of Hokuriku Agricultural Experiment Station has focused on the onset of internode elongation in normal paddy rice plants and classified the onset of internode elongation based on changes in the internode structure into two phases: the primary phase of internode elongation, which is the start of very short internode growth during the vegetative growth stage, and the secondary phase, which is the start of rapid and significant elongation during the reproductive growth stage (Suetsugu 1968). He also described that the onset of the second phase differs among rice varieties.

Later, research on the onset of internode elongation was conducted for deepwater rice. Deepwater rice has a remarkable ability to induce internode elongation in deepwater environments, sometimes elongating 20 cm or more in a single day. However, surprisingly, these deepwater rice plants drown if they do not reach the age at which internode elongation can occur. Therefore, the ability to elongate internodes from an early age (i.e., the early secondary phase proposed by Suetsugu) is an important trait for deepwater rice plants to survive in flooded environments. Dr. Inouye of Kyushu University revealed that the early onset of the secondary vegetative growth phase is strongly correlated with internode length in deepwater environments and proposed that LEI is one of the phenotypes representing internode elongation in deepwater rice plants (Inouye 1983). Furthermore, Dr. Inouye and colleagues investigated the relationship between GA and LEI by treating normal paddy rice and deepwater rice with GA and reported that GA treatment significantly reduced the LEI (induced early internode elongation) in deepwater rice, but that GA did not promote internode elongation in normal paddy rice (Inouye and Kim 1985). This result suggests that sensitivities to GA are higher in deepwater rice, and promote a lower LEI (early internode elongation).

As mentioned above, ethylene accumulates in normal paddy rice in a deepwater environment, and the ABA content, which negatively regulates internode elongation by acting antagonistically with GA, is markedly reduced in deepwater rice. On the other hand, the mechanism whereby ethylene signaling increases GA biosynthesis is lacking in normal paddy rice. Thus, the low amount of GA in normal paddy rice plants in a deepwater environment may be the reason for the lack of internode elongation. Therefore, we examined whether deepwater treatment with a GA solution could accelerate internode elongation by compensating for the low GA biosynthetic capacity of normal paddy rice plants. The results showed that deepwater treatment with GA further promoted internode elongation in deepwater rice plants compared to deepwater treatment without GA, whereas deepwater treatment with a GA solution did not induce internode elongation in normal rice plants. These results strongly suggest that an increase in the amount of GA in the internodes is insufficient for early internode elongation, and that increased sensitivity to GA is essential. Therefore, we hypothesized that the causal genes of the chromosome 3 and 12 QTLs that control LEI are factors that induce early internode elongation by increasing sensitivity to increased GA in deepwater rice during submergence. To examine this hypothesis, NIL3 and NIL12 lines were generated by introducing the QTL region of deepwater rice chromosomes 3 or 12 into the T65 genetic background. Treating these lines with GA induced early internode elongation in response to GA. In addition, an additive and promotive effect was observed in NIL3-12 in which the two regions were integrated into the T65 genetic background (Nagai *et al.* 2014, 2020). Based on these results, we hypothesized that the causal genes of the QTLs on chromosomes 3 and 12 in deepwater rice decreased the LEI (ie., promoted early internode elongation) by increasing GA sensitivity in response to increased GA in the deepwater environment.

Positional cloning of *qLEI3* on chromosome 3 identified a gene encoding a protein of unknown function, which we designated *ACCELERATOR OF INTERNODE ELONGATION 1* (*ACE1*) (Nagai *et al.* 2020). The *ACE1* of deepwater rice C9285 and normal paddy rice T65 were expected to encode different proteins because of a 1-bp insertion/deletion near the N-terminal region in the coding sequence. To evaluate which type is functional, we generated transgenic plants carrying *ACE1*-containing genomic regions and *ACE1* overexpressors. In both transgenic experiments, GA treatment induced significant internode elongation in plants transgenic for C9285-type *ACE1*, whereas transgenic rice plants carrying T65-type *ACE1* did not show internode elongation by GA treatment. These results indicate that in deepwater rice *ACE1* accelerates internode elongation, while *ACE1* of the T65 type represented a loss of function. Although no increase in mitotic activity occurred in the IM of C9285-type *ACE1* overexpressors under normal growth conditions, GA treatment activated cell division in the IM. On the other hand, GA did not activate cell division in the control plants. In addition, deepwater conditions as well as GA treatment induced *ACE1* expression in deepwater rice plants. We previously showed that deepwater rice accumulates GA during submergence through ethylene. Thus, the deepwater-dependent accumulation of GA may trigger *ACE1* expression in deepwater rice. Although the detailed function of ACE1 is unknown, these results suggest that *ACE1* is expressed in deepwater rice plants in deepwater environments due to increased GA levels, which increase GA sensitivity in the IM, activate cell division, and ultimately induce internode elongation. A gene called *FLOWERING PROMOTING FACTOR 1* (*FPF1*) has been reported as a rice *ACE1* homolog in *Arabidopsis* (Kania *et al.* 1997). Overexpression of *FPF1* in *Arabidopsis* results in early flowering. On the other hand, overexpression of *Arabidopsis* *FPF1* in rice did not induce internode elongation or flower development (Nagai *et al.* 2020), and overexpression of rice *ACE1* in *Arabidopsis* has little effect on internode elongation or flowering (unpublished). These results suggest that these genes are functionally different from each other. Future studies and clarification of the details of the molecular function of ACE1 will help us understand the mechanism of cell division maintenance in the IM.

We also performed positional cloning of *qLEI12* and identified a gene encoding a C2H2-type zinc-finger transcription factor as the causative gene and named it *DECELERATOR OF INTERNODE ELONGATION 1* (*DEC1*) (Nagai *et al.* 2020). As mentioned earlier, two QTLs, *qTIL12* and *qLEI12*, have been identified at the end of chromosome 12. The *SK* genes were identified as the causative genes for *qTIL12*, and *DEC1* was newly identified as the causative gene for *qLEI12*, indicating that the respective causative genes involved in TIL and LEI exist in this region. Gómez-Ariza *et al.* (2019) hypothesized that downregulation of the genes that maintain the vegetative phase triggers a switch to the reproductive phase in rice and they focused on genes whose expression is downregulated upon the transition to the reproductive phase. They identified *PREMATURE INTERNODE ELONGATION 1* (*PINE1*) in Nipponbare prior to our report of *DEC1*, which is the same gene as *DEC1*. Therefore, we will refer to this gene here as *PINE1*/*DEC1*. A comparison of the *PINE1*/*DEC1* sequences revealed the presence of three insertions/deletions and two amino acid substitutions in the C9285 and T65 PINE1/DEC1 amino acid sequences. We generated C9285 *DEC1-* and T65 *DEC1*-overexpressing plants to investigate the function of *PINE1*/*DEC1*. Interestingly, internode elongation was suppressed in both overexpressed plants. In contrast, the CRISPR-Cas9-induced *pine1*/*dec1* mutant in T65 promoted internode elongation under normal growth conditions, suggesting that *PINE1*/*DEC1* is an inhibitory regulator of internode elongation. The *PINE1*/*DEC1* gene was highly expressed near the IM in the internode of deepwater rice under normal growth conditions, but its expression was markedly reduced under deepwater conditions. *PINE1*/*DEC1* expression was also reduced by GA treatment in deepwater rice. However, the expression of *PINE1*/*DEC1* did not decrease in a deepwater environment or respond to GA treatment in normal paddy rice T65 and Nipponbare plants during the vegetative growth phase. There are multiple SNPs and indels between promoter regions of deepwater rice C9285 and T65. Therefore, these differences may lead to the different expression levels of DEC1 in C9285 and T65. As internode elongation was enhanced in the *dec1* mutant, we examined the activity of cell division at the internode. Cell division in the IM was activated in the *pine1*/*dec1* mutant without GA treatment. Furthermore, GA treatment induced an increase in the number of meristematic cells and expansion of the meristematic zone in the *pine1*/*dec1* mutant. These results suggest that PINE1/DEC1 is directly involved in cell division in the IM, and that reduced *PINE1*/*DEC1* expression in deepwater rice plants in a deepwater environment leads to the release of mitotic inhibition in the IM and the induction of internode elongation.

As mentioned above, normal paddy rice has a nonfunctional ACE1 protein due to a 1-bp insertion in the *ACE1* coding sequence. In addition, *PINE1*/*DEC1* is constitutively highly expressed during the vegetative growth phase. Therefore, internode elongation in normal paddy rice is suppressed. However, the expression level of *ACE1-LIKE1*, a homolog of *ACE1*, increased and the expression level of *PINE1*/*DEC1* decreased in normal paddy rice plants after the transition to the reproductive growth phase. Therefore, internode elongation is initiated by the transition to the reproductive growth stage in normal paddy rice plants. These studies of the genes responsible for the two QTLs regulating LEI have identified two novel factors involved in GA sensitivity in rice internode elongation and have revealed that internode elongation is regulated by a balance between ACE1 and PINE1/DEC1, two factors with opposing effects on the IM. In addition, overexpression of C9285-type *ACE1* in *Brachypodium distachyon*, barley, and sugarcane, as well as in rice, promotes internode elongation. In contrast, overexpressing *PINE1*/*DEC1* in barley suppresses internode elongation. These results suggest that the regulatory mechanism of internode elongation involving *ACE1* and *PINE1*/*DEC1* is shared across grass species.

## Application of three QTLs to produce flood-tolerant rice

**Fig. 6.**
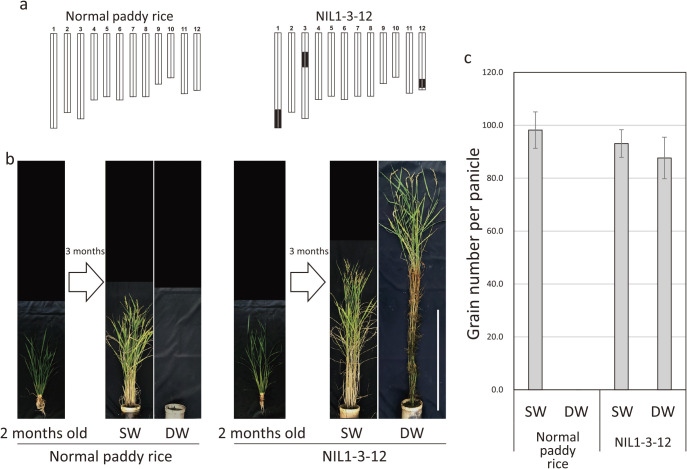
Evaluation of deepwater applicability of detected QTLs. a. Graphical genotype of normal paddy rice and NIL1-3-12. White and black bars represent genomic region of normal paddy rice and deepwater rice, respectively. b. Photographs of plants before and after deepwater treatment. SW represents the normal paddy field (shallow water) environment and DW represents the deepwater environment. DW treatment was continued for 3 months. Bar, 1m. c. Number of grains per panicle. Data are mean ± SD. Modified from Nagai *et al.* (2020).

We detected four QTLs that regulate internode elongation in deepwater rice plants during flooding and have identified the genes responsible for the QTLs. Therefore, we tested whether introducing these QTLs into normal paddy rice T65 would confer flood tolerance to T65. Through crossing and DNA marker selection, a QTL-accumulated line (NIL1-3-12) with four QTLs introduced in the T65 genetic background was generated (Fig. 6a). This line and parental line T65 were grown for 3 months in a flooded environment, and internode length and yield were determined. The T65 drowned in the flooded environment for 3 months (Fig. 6b, 6c). In contrast, NIL1-3-12 with the four QTLs induced internode elongation in response to flooding and we harvested seeds (Fig. 6b, 6c). These results indicate that these four QTLs play an important role in the adaptation of rice to flooded environments through internode elongation.

## Conclusion

**Fig. 7.**
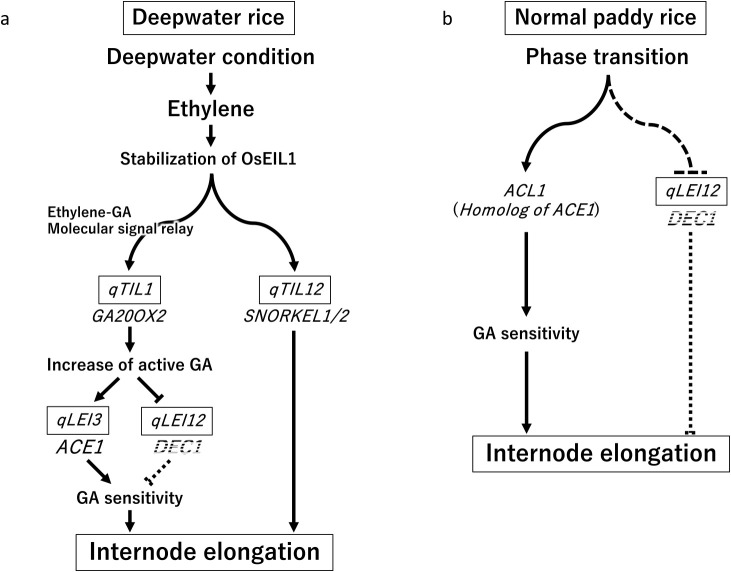
Regulatory mechanism of internode elongation by causal genes of QTLs. Signal pathway in internode elongation in deepwater rice under deepwater condition (a) and normal paddy rice in normal growth condition (b).

Deepwater rice plants overcome prolonged flooding conditions by acquiring a deepwater-dependent internode elongation ability. We detected the QTLs regulating internode elongation in deepwater rice in 2007, and we have identified the respective causal genes (Fig. 7a). The deepwater rice ethylene signaling factor OsEIL1 was stabilized by ethylene, accumulated under deepwater conditions, and *OsEIL1* expression was upregulated by binding to the promoter of *GA20OX2*, the causal gene of *qTIL1*. This pathway may function as an ethylene-GA molecular signaling relay. Deepwater rice possesses a highly enzymatically active form of GA20OX2, resulting in increased biosynthesis of active GAs (GA_1_ and GA_4_). The accumulated GAs upregulate the expression of *ACE1*, the causal gene of *qLEI3*, and ACE1 activates the IM with GA leading to internode elongation. Furthermore, GAs reduce the expression of the internode elongation repressor *PINE1*/*DEC1*, the causal gene of *qLEI12*, resulting in a decrease in the repressive capacity of PINE1/DEC1, which initiates internode elongation in deepwater rice. In addition, OsEIL1 binds to the promoter regions of *SK1* and *SK2*, the genes responsible for *qTIL12*, which promote internode elongation, to increase their expression. Although *ACE1* is defective in normal paddy rice, the expression of its homolog *ACL1* increases during reproductive growth, while the expression of *DEC1* decreases, inducing internode elongation (Fig. 7b). Identification and functional analysis of these genes have improved our understanding of the genetic mechanisms in addition to previous physiological findings of internode elongation in deepwater rice. However, these results provide only a part of the internode elongation mechanism. There are still unknown factors and missing links. Thus, further study is necessary to understand the mechanisms comprehensively.

Global warming since the latter half of the 20th century has resulted in floods causing damage to crops in recent years. Thus, clarification of the molecular mechanisms controlling internode elongation in deepwater rice plants will enable molecular breeding using marker selection and genome editing techniques to breed flood-tolerant rice plants. In addition, we expect that knowledge obtained on internode elongation in rice will be applied to flood-tolerant breeding of rice and other crops.

## Author Contribution Statement

K.N. and M.A. wrote the manuscript.
